# Pharmaceutical Development of Film-Coated Mini-Tablets with Losartan Potassium for Epidermolysis Bullosa

**DOI:** 10.3390/pharmaceutics14030570

**Published:** 2022-03-05

**Authors:** Valentinë Elezaj, Ard Lura, Luis Canha, Jörg Breitkreutz

**Affiliations:** 1 Institute of Pharmaceutics and Biopharmaceutics, Heinrich Heine University, 40225 Düsseldorf, Germany; valentine.elezaj@hhu.de (V.E.); ard.lura@hhu.de (A.L.); 2 Midas Pharma GmbH, 55218 Ingelheim, Germany; luis.canha@midas-pharma.com

**Keywords:** mini-tablet, paediatrics, losartan potassium, epidermolysis bullosa, rare disease, dry granulation, coating, stability

## Abstract

Epidermolysis bullosa is a genetically heterogenous skin fragility disorder with multiorgan involvement appearing already in newborn children. Severe progressive fibrosis follows skin blistering, mucosa lesions, and wound healing, favouring development of highly aggressive squamous cell carcinomas. Losartan potassium (LP) has been described to show positive effects; therefore, it was of clinical interest to develop 2 mm mini-tablets with LP for treatment of the affected children. Several challenges emerged during development: limited flowability and sticking to punches were observed in the first tableting experiments due to a high drug load, and a bitter taste of the LP was reported. Sticking to punches was reduced by using SMCC 50 and a combination of different lubricants; however, direct compression trials on a Korsch XM 12 rotary press were not successful due to compaction phenomena in the hopper. Thus, an intermediate dry granulation was successfully introduced. Two final formulations of the mini-tablets complied with the requirements of the *European Pharmacopoeia* regarding disintegration times (<15 min) and friability (<1.0%); mean tensile strengths amounted to about 1 MPa as a compromise between manufacturability and sufficient mechanical strength for further coating studies. The subsequent coating step succeeded delaying the initial drug release for more than 2 min. An acceptance value ≤15 was matched for the coated mini-tablets, and stability studies showed a promising shelf life.

## 1. Introduction

The development of mini-tablets is becoming increasingly important in the field of paediatric formulations. The Paediatric Regulation 1901/2006 was established in 2007 with the aim to promote the development of medicine for children. Further goals are appropriate authorization and more information on the application of medicines for paediatric patients, while not delaying authorization of medicinal products for adults [1,2]. In the *Report of the Informal Expert Meeting on Dosage Forms of Medicines for Children* published by the World Health Organization (WHO) in 2008, the use of oral solid dosage forms over liquids was suggested for children, and was further supported by scientific experts [3,4,5].

In this study, the main focus was to develop an oral solid dosage form to address the rare disease epidermolysis bullosa (EB) [6], which can appear already in newborn children. EB is an inherited skin fragility disorder characterised by genetic heterogeneity. This disease is associated with deficient epithelial cell adhesion, which can cause skin detachment and blistering. A differentiation is made between types, of which dystrophic epidermolysis bullosa (DEB) is one of the major groups. DEB is caused by mutations in the gene for collagen VII, an extracellular matrix adhesion protein involved in the adherence of the epidermis to the dermis. Various skin and organ manifestations that affect, for example the heart, liver, and kidneys, can be observed. Skin blistering can be locally limited or generalised, and can also affect the mucosa. Repeated inflammation, wounding, and scarring can result in fibrosis, with the risk of promoting further diseases such as squamous cell carcinoma, pseudosyndactyly, and oesophageal stenosis [7,8,9,10,11,12,13,14,15].

The drug losartan potassium (LP) was found to have beneficial effects in the treatment of this disease by decreasing TGF-β expression, the activity of which is increased in this skin disorder [7,8,9,10,11,12,13,14,15]. In the REFLECT trial with children of age 2 to 16 years with recessive DEB, the treatment with LP included an escalation period, a treatment period at a dosage of about 1.4 mg/kg body weight per day, and possibly a tapering phase [13].

For LP to be used in paediatric patients, an appropriate dosage form is required. Mini-tablets, defined as tablets with a size of 2–3 mm and a surface-to-volume ratio of at least 2 mm^−1^, represent a promising solid dosage form [16,17]. Various studies have indicated the acceptability and swallowability of single and multiparticulate 2 mm mini-tablets for the paediatric population; single-coated 2 mm mini-tablets were also tested [18,19,20,21]. On the basis of these studies, 2 mm mini-tablets were chosen as a promising solid dosage form, with the expectation of similar acceptability and swallowability. Good swallowability is an important issue, especially in cases of stenosis in the oesophagus and damage to the mucosa. Furthermore, dosing flexibility facilitates individual dosing by body weight [22,23].

To the best of our knowledge, currently there are no formulations available on the market containing losartan potassium that would be suitable at the required doses for the paediatric population when considering acceptability, swallowability, and dosing flexibility. Therefore, the aim of this study was to develop 2 mm mini-tablets with a relatively high drug load of 2.5 mg LP per mini-tablet. The first scope was the manufacturing by direct compression, as according to Manufacturing Classification System (MCS), which proceeds through Classes 1 to 3 (direct compression and dry and wet granulation), the number of manufacturing steps increases, possibly leading to greater costs [24]. If this was not successful, the next step would be intermediate dry granulation. From the beginning of the formulation development, a number of challenges had to be taken into account. On the one hand, chosen excipients had to be suitable for the paediatric patients. On the other hand, the chosen drug load of 2.5 mg in a mini-tablet of 6.5 mg was relatively high, corresponding to almost 40% (*w*/*w*) drug loading. This made the poor flowability of LP relevant to the production, as characteristics of the drug have an effect on producibility and the final dosage form [24]. Additionally, the bitter taste of LP had to be overcome; therefore, a film coating had to be applied, and dissolution studies had to be conducted to investigate if the initial burst in drug release could be reduced. In the early development phase, a STYL’One Evo compaction simulator (MEDELPHARM, France) was used to keep material consumption low, before tableting was transferred to an XM 12 rotary tablet press (Korsch, Germany). Stability studies were conducted in different conditions and through the use of various packaging options to critically examine the properties of the mini-tablets regarding appearance, content uniformity, dissolution, and purity.

## 2. Materials and Methods

### 2.1. Materials

Losartan potassium (Form I, Dr. Reddy’s Laboratories, Jinnaram Mandal, Telangana, India), silicified microcrystalline cellulose (PROSOLV^®^ SMCC 50, JRS Pharma, Rosenberg, Germany), copovidone (Kollidon^®^ VA 64 (Fine), BASF Pharma, Ludwigshafen, Germany), crospovidone (Kollidon^®^ CL, BASF, Ludwigshafen, Germany), dicalciumphosphate anhydrous (DI-CAFOS^®^ A 150, Chemische Fabrik Budenheim, Budenheim, Germany), lactose (Tablettose^®^ 80, Flowlac^®^ 100, MEGGLE, Wasserburg am Inn, Germany), isomalt (galenIQ^TM^ 721, BENEO-Palatinit, Mannheim, Germany), microcrystalline cellulose (VIVAPUR^®^ 102, JRS Pharma, Rosenberg, Germany), Ludipress^®^ (BASF Pharma, Ludwigshafen, Germany), magnesium stearate (Parteck^®^ LUB MST, Merck, Darmstadt, Germany), sodium stearyl fumarate (PRUV^®^, JRS Pharma, Rosenberg, Germany), talc (Talkum^®^ Pharma G, C.H. Erbslöh, Krefeld, Germany), HPMC (PHARMACOAT^®^ 603, Shin-Etsu Chemical, Tokyo, Japan), macrogol (PEG 6000, BASF Pharma, Ludwigshafen, Germany), methanol (HPLC grade, VWR Chemicals, Radnor, PA, USA), acetonitrile (HPLC grade, Honeywell, Charlotte, NC, USA), phosphoric acid (Merck, Darmstadt, Germany), monobasic potassium phosphate (Fisher Scientific, Waltham, MA, USA), disodium hydrogen phosphate anhydrous (AppliChem, Darmstadt, Germany), and sodium hydroxide solution 1 N (Merck, Darmstadt, Germany) were used.

### 2.2. Methods

#### 2.2.1. Particle Size and Polymorphism

The particle size of the LP was analysed in triplicate via laser diffraction (Mastersizer 3000, Malvern, Malvern, UK) at an air pressure of approximately 0.8 bar.

Granules obtained by dry granulation were analysed by dynamic image analysis (Camsizer XT, Retsch, Haan, Germany) with an X-Jet module at an dispersion pressure of about 30 kPa. The particle characteristic x_area_ (the equivalent diameter obtained by the area of particle projection) was evaluated. Samples were previously divided by an automatic sample divider (Retsch, Haan, Germany).

The polymorphic form of LP was investigated using X-ray powder diffraction (Rigaku Miniflex diffractometer, Rigaku Co., Tokyo, Japan) in θ/2θ geometry with CuKα X-radiation (λ = 1.54182 Å) at 40 kV and 15 mA. Measurements were conducted for the pure LP, crushed mini-tablets of formulations 10C and 11 (F 10C and F 11), and their physical mixtures to investigate if the polymorphic form changes after granulation and tableting processes. The 2θ range of 2–50° was scanned at a rate of 5°/min.

#### 2.2.2. Scanning Electron Microscopy

The morphology of the LP was analysed via scanning electron microscopy (SEM) using a Phenom G2 pro (Phenom World, Eindhoven, The Netherlands) at 5 kV with a 730-fold magnification. The sample was previously gold-sputtered at a layer thickness of 15 nm by using an MSC1T automatic sputter coater (LOT Quantum Design, Darmstadt, Germany).

#### 2.2.3. Flowability

Evaluation of the flow properties of the powder mixtures or granules was done by determination of the Hausner ratio in triplicate according to *Ph. Eur. 10* 2.9.34 and 2.9.36 [25,26].

#### 2.2.4. Tableting on Compaction Simulator

In development step I, direct compression of various formulations (Table 1) was performed on the STYL’One Evo compaction simulator (MEDELPHARM, Beynost, France). Previously, the powder mixtures were blended for 15 min in a Turbula^®^ mixer at 49 rpm (W.A. Bachofen, Muttenz, Switzerland), before the lubricants were added and mixed. For example, in the case of F 3 with the excipient SMCC 50 (Table 1), the addition of magnesium stearate followed 2 min of mixing, then talc was added for another 2 min of blending. Then, 2 mm concave EU-B 19-tip punches (Ritter Pharma, Stapelfeld, Germany) were used for tableting at a machine speed of 20% using one compression mode. Dies were filled manually, because currently no suitable feeding shoe for mini-tablet manufacturing is available. The target dose was 2.5 mg LP per mini-tablet (target mass: 6.5 mg). The aim was to produce mini-tablets at three different pressures: approximately 50, 100, and 150 MPa.

#### 2.2.5. Tableting on Rotary Tablet Press

In development step II, F 8 (Table 2), as the most promising formulation from preliminary studies on the compaction simulator, was tableted on the XM 12 rotary tablet press (Korsch, Berlin, Germany) with an EU-B/D mixed rotor equipped with a feeding shoe with two counterrotating paddles and six concave 2 mm EU-D 19-tip punches (Ritter Pharma, Stapelfeld, Germany). Tableting was performed at a turret speed of 30 rpm, and tableting pressures of 50, 100, and 150 MPa were targeted. The target mass was set to 6.5 mg per mini-tablet. For this purpose, F 8 (batch size: 1.2 kg and later repeated with approximately 2 kg) was mixed for 20 min in an LM 40 lab-scale blender (L.B. Bohle, Ennigerloh, Germany), then magnesium stearate (sieved at 315 µm) and subsequently talc (sieved at 125 µm) were added and blended for another 3 and 2 min, respectively. The feasibility of tableting F 9 (batch size: 1.2 kg) was tested under the same process conditions (Table 2).

In the course of development step III (Table 3), extragranular components were added to the granules of F 10A, 10B, and 10C obtained at different specific compaction forces (2, 4, and 6 kN/cm) and blended for 20 min, then magnesium stearate and talc were added and mixed for another 3 and 2 min, respectively. Batch sizes were around 2.6, 2.5, and 2.3 kg for F 10A, 10B, and 10C, respectively. The resulting blend was tableted on the rotary press at 20 and 30 rpm at different tableting pressures, and for a longer period at 100 MPa, to obtain a sufficient amount of mini-tablets for the following coating step. For F 11 (Table 3), using a batch size of 1.4 kg, the same procedure was applied, and tableting was performed at 20 rpm.

#### 2.2.6. Roll Compaction/Dry Granulation

For F 10, a 9.0 kg powder mixture, and for F 11, a 2.0 kg powder mixture composed of the intragranular components (Table 3) were mixed for 20 min at 30–34 rpm in the LM 40 lab-scale blender (L.B. Bohle, Ennigerloh, Germany); the LP was previously sieved at 710 µm for deagglomeration. Dry granulation was performed using a roller compactor (Mini-Pactor^®^, Gerteis Maschinen and Processengineering, Jona, Switzerland) with smooth rolls. The gap between the rolls was set at 2.0 mm, and the speed of the rolls was adjusted to 3 mm. For F 10A, 10B, and 10C, specific compaction forces of 2, 4, and 6 kN/cm, and for F 11, 5 kN/cm, were applied to produce ribbons, which were then granulated using a star granulator with a 1.0 mm sieve. 

#### 2.2.7. Film-Coating

Mini-tablets of the F 10C (tableting pressure around 100 MPa, 30 rpm) and F 11 (tableting pressure around 100 MPa, 20 rpm) batches were coated using a Mycrolab fluid bed coater (Hüttlin, Germany) via bottom spray. An inlet air volume of 13 m^3^/h, an inlet air temperature of 48 °C, and a pump rate of 1.3 g/min were chosen. The spray pressure amounted to 1.0 bar, and the microclimate was set to 0.15–0.25 bar. An aqueous solution of 12.0% hydroxypropylmethylcellulose (HPMC) was chosen for taste-masking purposes, taking into account the applicability in the paediatric population. We used 1.2% Macrogol 6000 as a plasticiser. The batch size of mini-tablets to be coated was approximately 50 g and the targeted polymer application was 10 mg/cm^2^, based on preliminary studies to achieve a sufficient delay of more than 2 min in release in the first minutes (data not shown).

#### 2.2.8. X-ray Micro-Computed Tomography (XµCT)

Coated mini-tablets of F 10C and 11 were measured using XµCT (CT alpha, Procon X-ray, Sarstedt, Germany). The film thickness was determined at three locations each at the cap and the wall height of each mini-tablet (a total of 6 locations per mini-tablet); three mini-tablets were examined together per batch and per run. Measurements were done at a voxel size of 3.5 µm with 1600 projections at a voltage of 80 kV and a current of 40 µA. For image reconstruction, the VG Studio software (Volume Graphics, Heidelberg, Germany) was used, and for determination of the film thickness by manual line drawing, the Avizo Fire 9 Software was applied (Thermo Fisher Scientific, Waltham, MA, USA).

#### 2.2.9. Disintegration

Disintegration studies were performed with six uncoated mini-tablets in a *Ph. Eur.* apparatus in 37 ± 2 °C demineralised water [27]. According to a modified method by Kleinebudde [28], single mini-tablets were placed in Plexiglas cylinders and covered with a mesh sieve of 710 µm at the top and bottom before they were placed in a *Ph. Eur.* disintegration apparatus. The cylinders were weighted with a hollow metal cylinder.

#### 2.2.10. Tensile Strength

For 10 uncoated mini-tablets of each batch, the height and diameter were analysed with a digital caliper (Mitutoyo Absolute, Kawasaki, Japan). For the measurement of the diametral breaking force, a TA.XT.plus texture analyser (Stable Microsystems, Godalming, UK) with a 5 mm flat punch was used at a test speed of 0.1 mm/s. The final tensile strengths were calculated with Equation (1) by Fell and Newton [29]. Tabletability profiles were obtained by plotting tensile strengths against the tableting pressure.

TS = (2 × F)/(π × d × h)(1)

where TS = tensile strength (MPa), F = breaking force (N), d = diameter (mm), and h = height (mm).

#### 2.2.11. Friability

Friability was determined according to *Ph. Eur. 10* 2.9.7 with a sample size of 6.5 g of uncoated mini-tablets [30]. A total of 100 rotations of the drum (Erweka^®^ TA 120, Langen, Germany) at a speed of 25 rpm were performed. Dedusting of the mini-tablets was done via an air-jet sieve 200 LS-N (Hosokawa Alpine AG, Augsburg, Germany) with a 125 µm sieve at 300–500 Pa for 2 min. The test was performed twice.

#### 2.2.12. Content Uniformity

Content uniformity was determined according to *Ph. Eur. 10* 2.9.40 [31]. First, 10 mini-tablets were dissolved individually in methanol and filtered through a 0.45 µm nylon filter (Macherey-Nagel, Düren, Germany). The content was measured with high-performance liquid chromatography (HPLC) system with UV–vis coupling (Hitachi VWR, Germany) and acceptance values (AV) were calculated using Equation (2):

(2) $$\mathrm{AV}=\left|M-\bar{X}\right|+k\times s$$

where:

M = a reference value dependent on $\bar{X}$ (%);
$\bar{X}$ = mean of the contents, expressed as percentage of label claim (%);
k = acceptability constant (2.4 for *n* = 10); and
s = standard deviation (%).

If the AV exceeded the limit of 15.0 for level 1 (L1), *Ph. Eur.* allowed the measurement of a further 20 single mini-tablets, which were not tested in this work.

For content determination, a Eurospher II C18 column (250 × 4.6 mm, 5 µm particle size) with a precolumn (Knauer, Berlin, Germany) was used. An isocratic method was applied; the mobile phase consisted of acetonitrile and a solution of potassium dihydrogen phosphate (pH 2.2., 5 mM) (45:55). The column temperature amounted to 35 °C, and the flow rate was 1.3 mL/min. A total of 10 µL of the sample was analysed at a wavelength of 220 nm following the parameters for the test of impurities in the *Ph. Eur. 10* monograph on losartan potassium [32]. Evaluation was carried out with the EZChrome Elite software (Agilent Technologies, Santa Clara, CA, USA).

#### 2.2.13. Dissolution Studies

Dissolution studies (*n* = 3) were conducted in a basket apparatus with a sample size of 10 mini-tablets per basket. As dissolution medium, 900 mL phosphate buffer pH 6.0 tempered at 37 ± 0.5 °C was chosen. This medium is recommended in *Ph. Eur.* 2.9.25 for the dissolution test for medicated chewing gums, and was therefore chosen to reflect the medium in the oral cavity for dissolution studies of the mini-tablets, which were film-coated for taste-masking purposes [33]. The rotational speed of the baskets was set to 60–65 rpm, and sampling was done manually. Samples were centrifuged (MiniSpin^®^ plus, Eppendorf, Hamburg, Germany) and then analysed via HPLC using the assay for content uniformity described in Section 2.2.12.

#### 2.2.14. Stability Studies

Stability studies were performed for the coated mini-tablets using the 10C and 11 batches. They were stored using different packaging materials (openly, in polyethylene bags, or in sealed aluminium foil) and at two different conditions, namely 40 °C/75% r.h. (available climatic chamber) and 25 °C/60% r.h. (desiccator, containing a saturated solution of sodium bromide, in a drying chamber tempered to 25 °C). The critical quality attributes (CQAs) of appearance, content, purity, and dissolution were investigated after 0, 3, and 6 months.

For the analysis of the purity, HPLC analysis was performed according to the USP 39 monograph on losartan tablets with a few modifications regarding the used column and the flow rate [34]. The same column as described in Section 2.2.12 was used. The column temperature was set to 25 °C, and the flow rate to 1.3 mL/min. A total of 10 µL of the sample was analysed at a wavelength of 250 nm. An overview of the used mobile phase is provided in Table S1. Suitability requirements according to the monograph were tested and met before commencing the analysis of the samples. For the preparation of the sample solution (0.25 mg/mL), 10 mini-tablets were transferred to a 100 mL volumetric flask that was then filled with solution A (Table S1), with use of intermediate ultrasonication if necessary. The solution was passed through a 0.45 µm nylon filter (Macherey-Nagel, Germany) before injection into the HPLC system. The impurities 1H- and 2H-dimer were detected by analysis of the system suitability solution, manufactured according to the USP 39 monograph. Additionally, the reference substance of the *European Pharmacopoeia* (CRS), namely the “Losartan for system suitability CRS”, contained the impurities J, K, L, and M (as labelled in the *Ph. Eur. 10* monograph on losartan potassium) and hence contains the two dimers, so they were identified additionally by analysis of this CRS substance in combination with the system suitability solution. If other impurities apart from the 1H- and 2H-dimer appeared in the course of stability studies of the mini-tablets that were also found in the chromatogram of this CRS substance, they were referred to as “other single impurities”. All other impurities were referred to as “unknown impurities”. The sum of total impurities comprised all the specified and unspecified impurities. Peaks of less than 0.1% were not regarded in the calculation, and therefore were not included in the sum of single, unknown, and total impurities. When unknown impurities overlapped with the main peak of losartan, the integration was done by the software at the minimum between the peaks. The acceptance criteria were adapted from the USP monograph and were not more than 0.5% each for 1H- and 2H-dimer, and not more than 1.0% for the sum of total impurities. These limits could be extended, as they could show the release specifications. The percentage of each impurity in the mini-tablets was calculated according to USP monograph shown in Equation (3):

R = (A_U_/A_S_) × (C_S_/C_U_) × 100 (3)

where:

R = resulting percentage of impurity (%);
A_U_ = area of each individual impurity from the sample solution;
A_S_ = area of losartan peak from the standard solution (2.5 µg/mL of CRS losartan potassium in solution A);
C_S_ = concentration of CRS losartan potassium in standard solution (=2.5 µg/mL); and
C_U_ = nominal concentration of losartan potassium in the sample solution (=250 µg/mL).

## 3. Results and Discussion

### 3.1. Development Step I

**Figure 1:**
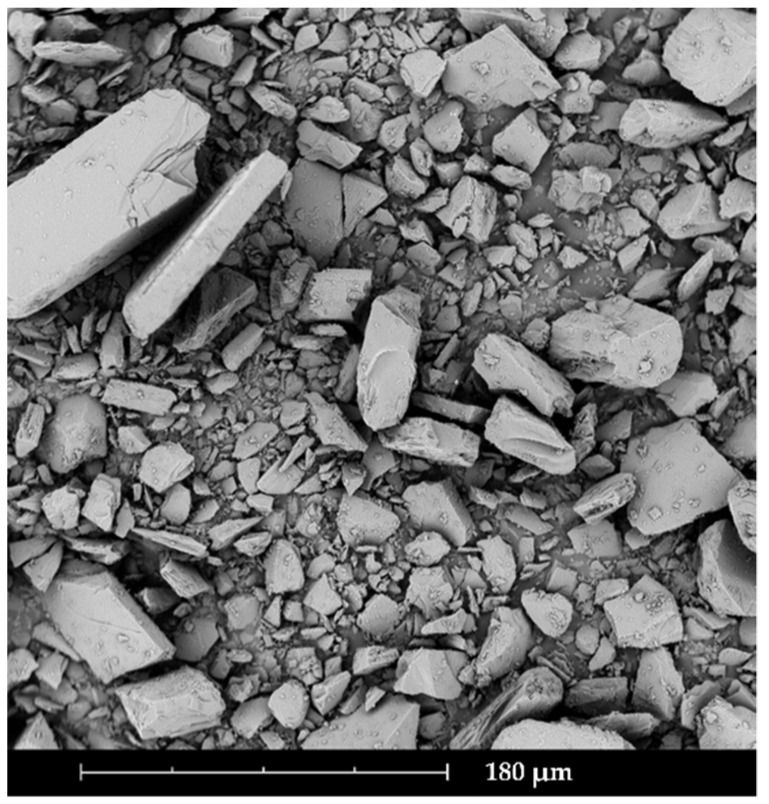
SEM image of losartan potassium with 730-fold magnification.

The LP showed a relatively broad particle size distribution, with a mean D_10_ of 4 µm, D_50_ of 29 µm, and D_90_ of 97 µm. The SEM image of the LP (Figure 1) revealed a block-shaped morphology, and the particles were edgy and irregular. The mean Hausner ratio of LP amounted to 1.50 ± 0.01 (mean ± SD), which described a very poor flowability (*Ph. Eur. 10*).

**Figure 2:**
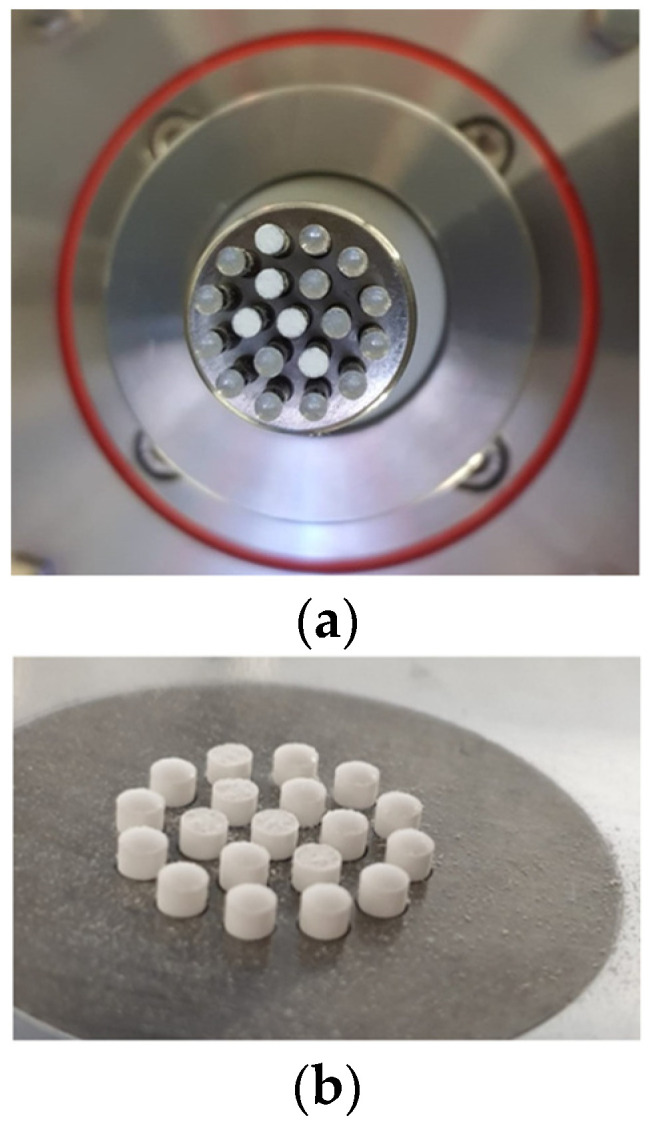
(**a**) Sticking on punches (upper punch displayed), leading to (**b**) damaged mini-tablets of F 6 with SMCC 50 and 3% SSF.

In development step I, different excipients were tested (Table 1) and tableted on the STYL’One Evo compaction simulator. At the very beginning, it became apparent that another challenge had emerged with the sticking tendency of LP. Various lubricants, including magnesium stearate, sodium stearyl fumarate (SSF), talc, and combinations thereof were tested to minimise the sticking tendency (Table 1). However, there were signs of sticking on the upper and lower punch depending on the formulation, and sometimes whole parts of the mini-tablets remained on the punches, as can be seen in Figure 2 for F 6 including SMCC 50 and 3% (*w*/*w*) SSF. In some cases, the mini-tablets could not even be removed from the dies without causing any damage to the mini-tablets. Intermediate cleaning of the punches with an organic solvent did not lead to a reduction in the adhesion. Environmental conditions, such as temperature and humidity, may have had an influence on the sticking degree [35,36], but the tableting studies were carried out in a climatic room at 21 °C and 45% r.h., and should therefore have had no influence on the adhesion tendency. An increase in tableting pressure and a decrease in the tableting speed also has been reported to aid in a reduction in the sticking tendency [35,37]; however, the tableting speed was not reduced, as this would not present the most satisfactory solution in industrial manufacturing with respect to total manufacturing time. The tableting pressure could not always be increased as an option to reduce the sticking tendency, because in some cases, the ejection forces were already increased at lower pressure, and for safety reasons regarding the punches, tableting pressure was not further elevated. F 3 with SMCC 50, 2% (*w*/*w*) magnesium stearate, and 1% (*w*/*w*) talc proved to work on the compaction simulator regarding a reduction in the sticking tendency. In further preparatory studies, in which the amount of LP per mini-tablet was reduced, the amount of lubricant could be decreased in terms of sticking to the punches, indicating an impact of the drug load on the sticking tendency (data not shown). The observed sticking and picking phenomena for the LP were coherent with findings in the literature [38]. SMCC is a coprocessed excipient composed of microcrystalline cellulose and silicon dioxide with reported properties of antiadherence [39,40,41] and lower lubricant sensitivity compared to MCC [39,40,42,43,44,45].

### 3.2. Development Step II

**Figure 3:**
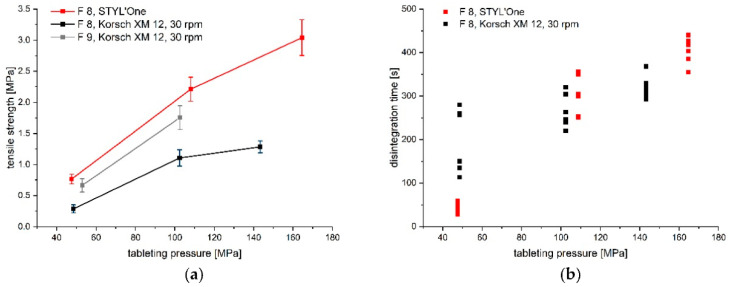
Comparison of (**a**) tabletability (mean ± CI (α = 0.05), *n* = 10) and (**b**) disintegration times (*n* = 6) of mini-tablets of F 8 produced on the STYL’One and on the Korsch XM 12; (**a**) also shows the tabletability (mean ± CI (α = 0.05), *n* = 10) of F 9 produced on the Korsch XM 12.

The most promising F 8, found in tableting studies on the STYL’One Evo in development step I, was tableted on the Korsch XM 12 rotary tablet press. The tabletability plot and disintegration times are displayed in Figure 3 together with the results obtained on the compaction simulator for comparison purposes. The disintegration times were below 15 min at all tableting pressures, and hence complied with the *Ph. Eur.* requirements. It was noticeable that the mean tensile strengths at the respective tableting pressures were significantly lower for the formulation tableted on the rotary press, achieving around 1.1 MPa at a tableting pressure of 100 MPa, compared to 2.2 MPa at about 110 MPa on the STYL’One. One opportunity for a reduction in the tensile strength was the high amount of lubricants used in the formulation, possibly in combination with a potential overlubrication during the residence time in the forced feeder, as shear stresses were continuously exerted by the two integrated paddles, influencing the distribution of the lubricant particles [46]. However, it had to be taken into account that the Korsch XM 12 simulation profile was not applied on the compaction simulator, because it was used for screening purposes only. Therefore, tableting on the rotary press was repeated with F 9; i.e., adding 1% (*w*/*w*) instead of 2% (*w*/*w*) magnesium stearate. Indeed, the mean tensile strength increased to 1.7 MPa at a tableting pressure of 100 MPa, indicating the influence of the lubricant on the tabletability. However, high ejection forces were the limiting factor for this formulation, and also prevented an increase in the tableting pressure. Therefore, F 8 was used for further studies, and the batch size was increased to 2 kg to test mini-tablet production at higher batch sizes. However, an increase in the batch size led to compaction in the hopper above the feed mechanism, which made further tableting no longer feasible, as the dies were no longer filled accurately (Table S2). We consequently concluded that, based on the MCS [24], the aim was now to implement an intermediate dry-granulation step representing development step III.

### 3.3. Development Step III

**Figure 4:**
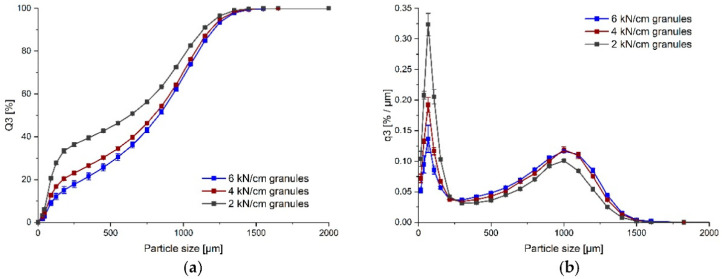
(**a**) Cumulative and (**b**) density particle size distribution (PSD) of granules (F 10) obtained at different specific compaction forces (2, 4, and 6 kN/cm); mean ± SD, *n* = 3.

**Figure 5:**
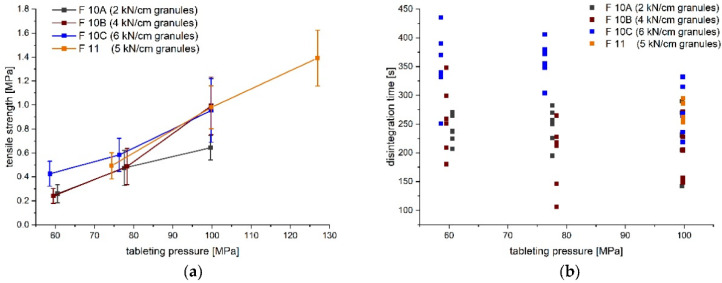
Comparison of (**a**) tabletability (mean ± CI (α = 0.05), *n* = 10) and (**b**) disintegration times (*n* = 6) of mini-tablets of F 10A, 10B, 10C, and 11.

F 10 was selected based on previous direct compression experiments, hence SMCC 50 was chosen again in combination with the same amount of lubricants due to the risk of sticking. In order to further increase the tensile strength, copovidone was also added as a dry binder to the formulation. The degree of the specific compaction force had an impact on the amount of the fine fraction. The fine fraction was reduced with increasing specific compaction force, shown as a shift to the right in the cumulative distribution curve and a decrease in the peak in the bimodal density distribution (Figure 4). The granules obtained at 6 kN/cm had a mean D_50_ of 832 µm and a D_90_ of 1204 µm (Table S3). With an increasing specific compaction force, the Hausner ratio tended to decrease, which may have been due to the reduction in the fine fraction. The granules obtained at 6 kN/cm showed passable flowability according to *Ph. Eur. 10*. (Table S3). Previous dry granulation prevented a comparable compaction in the hopper above the feed mechanism with the batch size used as it was observed during direct compression experiments. The tabletability plot of the resulting mini-tablets of the 10A, 10B, and 10C batches revealed, for the latter two, a mean tensile strength of about 1 MPa at a tableting pressure of approximately 100 MPa, as a compromise between the mechanical strength of the mini-tablets and the level of ejection force (Figure 5). The disintegration times of all mini-tablet batches were below 15 min, and hence complied with the limit for uncoated tablets in *Ph. Eur. 10*. The slight trend towards higher disintegration times with decreasing tableting pressure for F 10A, B, and C may have been due to the tableting procedure, because the mini-tablets were first tableted at 100 MPa for a longer period, before the tableting pressure was then decreased to obtain the tabletability plot, so the higher residence time in the forced feeder in combination with high amount of lubricants may have been the reason. The amount of lubricants was kept the same for the direct compression experiments; however, the specific surface of the granules was reduced compared to the DC preparation due to particle size enlargement. Still, this quantity appeared to be necessary so that the ejection forces did not increase.

Based on this, another granulation and tableting study was carried out using dicalcium phosphate (DCP) as a brittle excipient that is less sensitive to lubrication (F 11) [46] to investigate if the tensile strengths could be increased. Roll compaction was performed at a specific compaction force of 5 kN/cm, resulting in similar particle size distributions compared to the 6 kN/cm granules of F 10C; the mean D_10_ was 94 ± 5 µm, D_50_ 825 ± 19 µm, and D_90_ was 1193 ± 1 µm (mean ± SD, *n* = 3). Different specific compaction forces were not tested, but a reasonable force was selected based on the first granulation study in order to first investigate the feasibility of tableting, since the formulation was changed. Figure 5 displays similar tensile strengths at 100 MPa for F 11; a tableting pressure of about 130 MPa led to a mean tensile strength of approximately 1.4 MPa. The disintegration times of the mini-tablets at 100 MPa are displayed in Figure 5; they also complied with the set limits in *Ph. Eur. 10*.

In addition to the tensile strength, friability also was an essential factor to be determined with regard to further coating processes. The friability of mini-tablets of F 10A was not determined, because it was possible to visually see their low mechanical strength. The friability of each of the mini-tablets of the other formulations (all tableted at around 100 MPa) was below the recommended limit of 1.0% for uncoated tablets in the *Ph. Eur.* Mini-tablet batch F 10B showed a friability of 0.5 and 0.6% (*n* = 2), F 10C of 0.1 and 0.2% (*n* = 2) and F 11 of 0.3 and 0.4% (*n* = 2). Since the surface-to-volume ratio for the mini-tablets was higher compared to conventionally sized tablets [16], the recommended limit could be re-evaluated and possibly increased for this dosage form. Based on the results for the particle size distribution of the granules, as well as the tensile strength and friability of the mini-tablets, F 10C mini-tablets tableted at 100 MPa were selected for a further coating step. In the case of F 11, mini-tablets produced at 100 MPa were chosen for comparison purposes.

X-ray powder diffraction measurements revealed similar patterns in the uncoated mini-tablets of F 10C and F 11 compared to their respective physical mixtures, indicating no polymorphic transition after the granulation and tableting processes (data not shown). The pattern of LP showed a similar pattern to Form I as described by Raghavan et al. [47].

**Figure 6:**
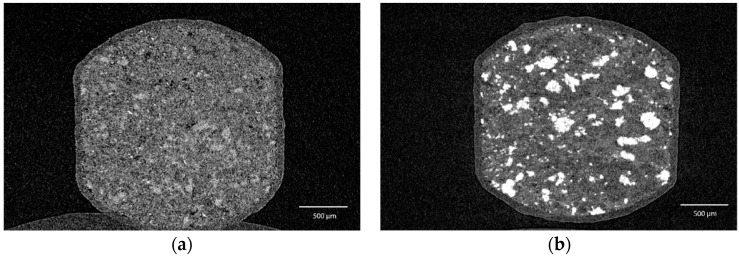
The µCT images of coated mini-tablets of (**a**) F 10C and (**b**) F 11.

Coating of both mini-tablet batches was feasible at the lab scale. The polymer application of 10 mg/cm^2^ was regarded as sufficient regarding the delay in drug release in the first minutes, based on preliminary studies (data not shown). HPMC was chosen as a coating polymer suitable for the paediatric population. Figure 6 shows µCT images of a mini-tablet of F 10C (a) and of 11 (b), indicating an even and intact film. From these images, the coating thickness was determined at three locations each at the cap and the wall height on three mini-tablets per batch. For mini-tablets of F 10C, the mean thickness at the cap and the wall height were 76.2 ± 7.5 µm and 78.7 ± 5.4 µm (mean ± SD), respectively. For mini-tablets of F 11, the mean thickness amounted to 70.4 ± 4.2 µm and 69.2 ± 3.4 µm at the cap and the wall height, respectively.

**Figure 7:**
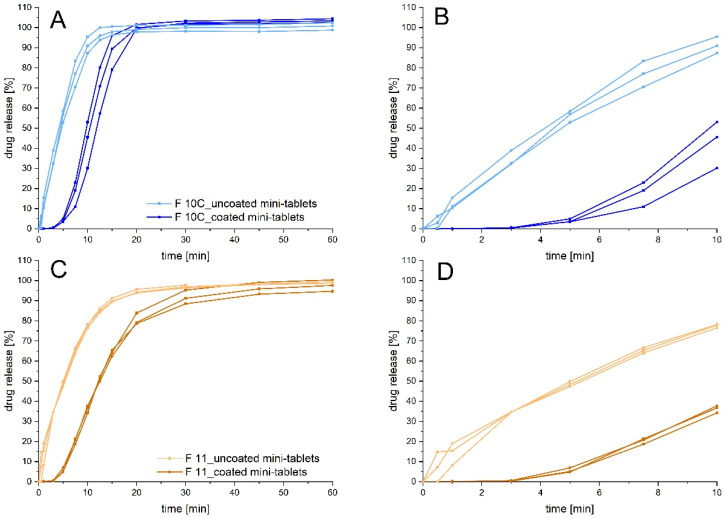
Dissolution profiles of coated and uncoated mini-tablets of F 10C (**A**) and 11 (**C**) and the respective magnification of the profiles in the first minutes (**B**,**D**); 37 ± 0.5 °C, medium: phosphate buffer pH 6.0; *n* = 3.

Final dissolution studies to compare the coated and uncoated mini-tablets (Figure 7) clearly showed that the uncoated mini-tablets had an initial burst in drug release in the first minutes, when the mini-tablet might be still in the mouth, causing an unpleasant taste. Coated mini-tablets, in contrast, showed a delay in drug release for more than 2 min. Still, the coated mini-tablets were an immediate release dosage form, since about 80% of drug release was obtained within about 20 or 30 min.

The content of the coated mini-tablets and the resulting acceptance value were determined for both formulations. For F 10C, a mean content of 97.1 ± 5.2%, and for F 11 of 95.6 ± 2.6% (mean ± SD, *n* = 10) resulted, corresponding to an AV of 14 and 9, respectively, and hence below the limit of 15 (*Ph. Eur. 10*).

**Figure 8:**
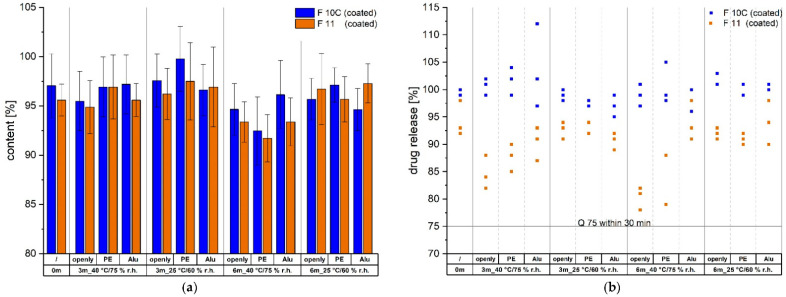
(**a**) Content (mean ± CI (α = 0.05), *n* = 10) and (**b**) drug release in % at 30 min (*n* = 3) of coated mini-tablets (F 10C and F11) stored at 40 °C/75% r.h. and 25 °C/60% r.h. in different packaging conditions (openly, in polyethylene bags (PE), and in sealed aluminium foil (Alu)). Selected timepoints: 0 months (0 m), 3 months (3 m), and 6 months (6 m) of storage.

The characterizations of the uncoated and coated mini-tablets were followed by a stability study of the final coated mini-tablets (F 10C and F 11). Figure 8 gives an overview of the content and drug release in % after 30 min of dissolution in storage under two different conditions and in various packaging conditions. The content did not change significantly over the storage period, but there was a slight trend of decline after 6 months in the stress conditions of 40 °C/75% r.h. when the mini-tablets were stored openly or in PE bags. Drug-release data displayed that generally, the drug release at 30 min was lower for the mini-tablets of F11 compared to those of F 10C. Values for F 10C scattered around 95% and 105%, and did not show a decreasing trend after exposure to stress conditions (40 °C/75% r.h.) for 6 months. In comparison, stress conditions seemed to have an influence on the mini-tablets of F 11, as after 3 months, the drug release decreased with open or PE packaging, and after 6 months with open packaging, it was even observed that after 30 min, only 78% to 82% was released compared to the initial timepoint with a drug release of 92% to 98%, and hence came close to the set limit of Q 75% within 30 min, which is stated in the USP 39 monograph on losartan tablets (the dissolution testing in the USP differed from the used dissolution setup). Storage in sealed aluminium foil prevented a comparable decline in drug release, so that 91% to 98% was released within 30 min. Storage at 25 °C/60% r.h. did not lead to a similar drop in drug release. The results of the purity analysis of the respective mini-tablets are listed in Tables S4 and S5. After 6 months, single impurities were detectable, and the sum of unknown impurities also increased especially during storage in stress conditions, but the acceptance criteria from the USP 39 monograph on losartan tablets were not exceeded (not more than 0.5% each for 1H- and 2H-dimer, and not more than 1.0% for the sum of total impurities). The mini-tablets of both batches had a white to yellowish appearance, and under stress conditions with open and PE packaging, the mini-tablets showed a yellow colour in comparison.

## 4. Conclusions

We successfully produced 2 mm mini-tablets with a high drug load of losartan potassium (2.5 mg per mini-tablet) after intermediate dry granulation; this is of clinical interest with regard to the treatment of the rare disease epidermolysis bullosa. Two formulations were dry-granulated and tableted on an XM 12 rotary tablet press, indicating that changes in the formulation still allowed successful tableting, implying a certain flexibility. Coating of the mini-tablets succeeded at the laboratory scale, and dissolution studies revealed an initial delay in the release of more than 2 min compared to the initial burst of uncoated mini-tablets, which would evoke an unpleasant bitter taste in the mouth. The results from the stability studies were promising for the observed time period of 6 months at 25 °C/60% r.h. and 40 °C/75% r.h. regarding the appearance of impurities. Dissolution should be monitored closely with regard to compliance of shelf-time specifications in the case of F 11, as the storage in stress conditions revealed a decrease in drug release at the dissolution timepoint of 30 min. These findings underlined the relevance of appropriate packaging. The next step would be the transfer to industrial production to verify the feasibility of manufacturing of the mini-tablets and subsequent film-coating at higher scales.

This study highlighted the potential challenges during formulation development of mini-tablets, in which compromises sometimes have to be made between the manufacturability and required ranges of critical quality attributes.

A continued focus will be placed on systematic investigations of direct tableting of formulations containing losartan potassium (with an alternative dosage) being transferred by compaction simulations from the STYL’One Evo to the XM 12 rotary press and subsequent scale-up procedures on the XM 12 to monitor predefined critical quality attributes. In cases of lubricant sensitivity and a resulting decrease in tensile strengths or elevations in disintegration times, external lubrication could be considered.

## 5. Patents

As a result of this work, a patent with the application number EP21172815.9 and title “Minitablette enthaltend Losartan für pädiatrische Anwendungen” (7 May 2021) was filed.

## Figures and Tables

**Table 1:** Overview of the main formulations of development step I. LP = losartan potassium; MgSt = magnesium stearate; SSF = sodium stearylfumarate.

Formulation	Material	Portion (% *w*/*w*)
1	LP	38.5
	Filler A–E ^1^	60.5
	MgSt	1.0
2	LP	38.5
	Filler A–E ^1^	59.5
	MgSt	2.0
3	LP	38.5
	Filler A–E ^1^	58.5
	MgSt	2.0
	talc	1.0
4	LP	38.5
	Filler A–E ^1^	60.5
	talc	1.0
5	LP	38.5
	Filler A–E ^1^	59.5
	MgSt	1.0
	talc	1.0
6	LP	38.5
	Filler A–E ^1^	58.5
	SSF	3.0
7	LP	38.5
	Filler A–E ^1^	57.5
	SSF	3.0
	talc	1.0

^1^ Each formulation was tested with each of the following fillers individually using the respective portion listed in Table 1: A (galenIQ^TM^ 721); B (Flowlac^®^ 100); C (Vivapur^®^ 102/Tablettose 80^®^ 10:90); D (PROSOLV^®^ SMCC 50); E (Ludipress^®^).

**Table 2:** Overview of formulations of development step II. LP = losartan potassium; MgSt = magnesium stearate; SMCC = silicified microcrystalline cellulose.

Formulation	Material	Portion (% *w*/*w*)
8	LP	38.5
	SMCC 50	58.5
	MgSt	2.0
	Talc	1.0
9	LP	38.5
	SMCC 50	59.5
	MgSt	1.0
	Talc	1.0

**Table 3:** Overview of formulations of development step III. LP = losartan potassium; MgSt = magnesium stearate; SMCC = silicified microcrystalline cellulose.

Formulation	Material	Intra-/Extragranular	Portion (% *w*/*w*)
10A, 10B, 10C ^1^	LP	Intra	38.5
	SMCC 50	Intra	51.5
	Copovidone ^2^	Extra	5.0
	Crospovidone	Extra	2.0
	MgSt	Extra	2.0
	Talc	Extra	1.0
11	LP	Intra	38.5
	SMCC 50	Intra	32.8
	DCP	Intra	19.0
	Copovidone ^3^	Intra	4.8
	Crospovidone	Extra	2.0
	MgSt	Extra	2.0
	Talc	Extra	1.0

^1^ F 10A, 10B, and 10C represent the same composition but differed in the specific compaction force used to prepare the granules before tableting (A: 2 kN/cm; B: 4 kN/cm; C: 6 kN/cm); ^2^ Kollidon^®^ VA 64 was used; ^3^ Kollidon^®^ VA 64 Fine was used.

## Data Availability

The data from this study are available in this paper and in the Supplementary Materials.

## Supplementary Materials

The following supporting information can be downloaded at: https://www.mdpi.com/article/10.3390/pharmaceutics14030570/s1, Table S1: Composition of mobile phase for purity analysis via HPLC, Table S2: Flow properties of F 8 and its single components (mean ± sd, *n* = 3, LP = losartan potassium, SMCC = silicified microcrystalline cellulose), Table S3: Particle size characterization and Hausner ratio of granules (F 10A, 10B, 10C) (mean ± sd, *n* = 3), Table S4: Purity of the coated mini-tablets (F 10C) stored at 40 °C/75% r.h. and 25 °C/60% r.h. in different packaging conditions (openly, in polyethylene bags (PE), and in sealed aluminium foil (Alu)). For better overview, only results at the initial timepoint and after 6 months of storage are shown (3rd-month timepoint not shown) (MT = mini-tablets, ND = not detected, <0.1% = impurities detected but not included in calculations), Table S5: Purity of the coated mini-tablets (F 11) stored at 40 °C/75% r.h. and 25 °C/60% r.h. in different packaging conditions (openly, in polyethylene bags (PE), and in sealed aluminium foil (Alu)). For better overview, only results at the initial timepoint and after 6 months of storage are shown (3rd-month timepoint not shown) (MT = mini-tablets, ND = not detected, <0.1% = impurities detected but not included in calculations).
