# Supplementary material for: Pharmaceutical Development of Film-Coated Mini-Tablets with Losartan Potassium for Epidermolysis Bullosa
Source: Pharmaceutics. 2022 Mar 5;14(3):570. doi: 10.3390/pharmaceutics14030570 (PMC8955998; doi:10.3390/pharmaceutics14030570)
Supplement: Supplementary file 1 [file pharmaceutics-14-00570-s001.zip › pharmaceutics-1546654-supplementary.pdf]

pharmaceutics

MDPI

Supplementary Material

Pharmaceutical Development of Film-coated Mini-tablets with Losartan Potassium for Epidermolysis Bullosa

Valentinë Elezaj, Ard Lura, Luis Canha and Jörg Breitkreutz

Table S1. Composition of mobile phase for purity analysis via HPLC

Mobile phase			
buffer	1.25 mg/mL potassium dihydrogen phosphate and 1.5 mg/mL di-sodium hydrogen phosphate in water (pH approximately 7.0)		
solution A	acetonitrile and buffer (15:85)		
solution B	acetonitrile		
gradient elution	**time [min]**	**solution A [%]**	**solution B [%]**
	0	80	20
	10	40	60
	11	80	20
	20	80	20

Table S2. Flow properties of F 8 and its single components, mean ± sd, n=3, LP = losartan potassium, SMCC = silicified microcrystalline cellulose

Material/powder	Hausner ratio	Flowability (Ph.Eur. 10)*
F 8	1.35 ± 0.01	poor
LP	1.50 ± 0.01	very poor
SMCC 50	1.27 ± 0.02	passable

*Rating by mean of Hausner ratio

Table S3. Particle size characterization and Hausner ratio of granules (F 10A, 10B, 10C), mean ± sd, n=3

	D_{10} [µm]	D_{50} [µm]	D_{90} [µm]	Hausner ratio	Flowability (Ph. Eur. 10)*
**F 10A (2 kN/cm)**	59 ± 1	634 ± 12	1135 ± 4	1.41 ± 0.01	poor
**F 10B (4 kN/cm)**	75 ± 1	799 ± 8	1183 ± 4	1.35 ± 0.02	poor
**F 10C (6 kN/cm)**	101 ± 13	832 ± 12	1204 ± 4	1.33 ± 0.02	passable

*Rating by mean of Hausner ratio

Table S4. Purity of the coated mini-tablets (F 10C) stored at 40 °C / 75 % r.h. and 25 °C / 60 % r.h. in different packaging conditions (openly, in polyethylene bags (PE) and in sealed aluminium foil (Alu)). For better overview only results at initial timepoint and after 6 months storage are shown (3rd month timepoint not shown), MT = mini-tablets, ND = not detected, < 0.1 % = impurities detected but not included in calculations

**Coated MT of F 10 C**		**6 months 25 °C / 60 % r.h.**			**6 months 40 °C / 75 % r.h.**		
**Purity**	**Initial**	**Openly**	**PE**	**Alu**	**Openly**	**PE**	**Alu**
1H Dimer	ND	ND	ND	ND	< 0.1 %	< 0.1 %	< 0.1 %
2H Dimer	ND	ND	ND	ND	ND	ND	< 0.1 %
Other single impurities	ND	ND	ND	ND	< 0.1 %	< 0.1 %	ND
Sum of unknown impurities	ND	< 0.1 %	0.10 %	< 0.1 %	0.11 %	0.13 %	0.13 %
Sum of total impurities	ND	< 0.1 %	0.10 %	< 0.1 %	0.11 %	0.13 %	0.13 %

Table S5. Purity of the coated mini-tablets (F 11) stored at 40 °C / 75 % r.h. and 25 °C / 60 % r.h. in different packaging conditions (openly, in polyethylene bags (PE) and in sealed aluminium foil (Alu)). For better overview only results at initial timepoint and after 6 months storage are shown (3rd month timepoint not shown), MT = mini-tablets, ND = not detected, < 0.1 % = impurities detected but not included in calculations

**Coated MT of F 11**		**6 months 25 °C / 60 % r.h.**			**6 months 40 °C / 75 % r.h.**		
**Purity**	**Initial**	**Openly**	**PE**	**Alu**	**Openly**	**PE**	**Alu**
1H Dimer	ND	< 0.1 %	< 0.1 %	< 0.1 %	< 0.1 %	< 0.1 %	< 0.1 %
2H Dimer	ND	< 0.1 %	< 0.1 %	ND	< 0.1 %	< 0.1 %	< 0.1 %
Other single impurities	ND	ND	ND	ND	< 0.1 %	< 0.1 %	< 0.1 %
Sum of unknown impurities	< 0.1 %	< 0.1 %	< 0.1 %	< 0.1 %	< 0.1 %	0.10 %	0.12 %
Sum of total impurities	< 0.1 %	< 0.1 %	< 0.1 %	< 0.1 %	< 0.1 %	0.10 %	0.12 %
